# Qualitative Evaluation of a Garden-Based Healing and Learning Program for Young Adults with Intellectual Disabilities

**DOI:** 10.3390/ijerph22020206

**Published:** 2025-01-31

**Authors:** Dohun Kim, Eunyeong Park, Hojun Yun, Yumi Baek, Hyeyoung Jin, Hyeryeong Cho

**Affiliations:** 1 Department of Research, Landscape Yeoleum, 65, Poeun-ro 2ga-gil, Mapo-gu, Seoul 04026, Republic of Korea; searz0921@gmail.com (D.K.); hjyun@yeoleum.co.kr (H.Y.); 2 Department of Environmental Landscape Architecture, Joongbu University, 305, Dongheon-ro, Deogyang-gu, Goyang 10279, Republic of Korea; eypark@joongbu.ac.kr; 3 Department of Educational Counseling Psychology, Joongbu University, 305, Dongheon-ro, Deogyang-gu, Goyang 10279, Republic of Korea; edubym@jbm.ac.kr; 4 Department of Garden and Education, Korea National Arboretum, 509, Gwangneungsumogwon-ro, Soheul-eup, Pocheon 11186, Republic of Korea; jinhye0@nate.com

**Keywords:** garden therapy, participant observation method, young adults with intellectual disabilities (YAwID), garden-based learning, healing gardens, place and health

## Abstract

This study investigated the impact of garden-based learning on young adults with intellectual disabilities (YAwID). Since YAwID often experiences difficulties collecting information, experiencing situations, and making decisions independently, we developed and implemented a customized learning program for them and analyzed its impact. This program was devised specially to help YAwID utilize the garden’s resources properly. The findings showed that garden-based learning generated specific changes in the knowledge, skills, and attitudes of YAwID, such as emotional healing to recover psychological stability, social healing through social relationships, physical healing through new activities, and independent healing through individualized plans. Participants experienced sympathizing with others and maintaining positive relationships and obtained the knowledge, skills, and attitudes necessary for behaving responsibly through gardening. Garden-based learning utilizing flowers and plants enhanced participants’ physical and mental health, improved their functioning, and helped them adapt to the environment and integrate into society. Our program can be considered a form of vocational training by which people with disabilities can enhance their employability by learning gardening skills, promoting their participation in society, and improving their quality of life. Hence, garden-based learning may transform people’s perception of disability and help people with disabilities respond to challenges.

## 1. Introduction

### 1.1. Overview

Disability denotes a physical or mental impairment that restricts daily living activities [1]. Intellectual disabilities are defined by incomplete or stalled cognitive development, significantly hindering daily functioning [2]. Individuals with intellectual disabilities exhibit diminished cognitive, linguistic, physical, and social capabilities. Such phenomena are evolving into significant social concerns. Recent years have seen the implementation of an integrative approach to address the physical and mental health needs of individuals with intellectual disabilities. This includes physiotherapy [3], psychotherapy [4], and effective communication strategies [5], all of which are crucial for improving the quality of life for these individuals. In terms of specific practices, research has demonstrated that leisure activities promote social competence [6], and gardening improves psychological and social wellbeing, as well as occupational performance [7,8,9,10,11]. This multidisciplinary study proves the beneficial effects of garden therapy.

Individuals with intellectual disabilities face the primary challenge of engaging as active members of society, which often leads to social isolation and loneliness [12]. This indicates that leading a conventional life as a community member presents significant challenges. Prior studies have predominantly concentrated on children, adolescents, and adults. While adults have found their place in society, younger generations are ill-prepared to deal with social contact. To enable individuals with intellectual disabilities to develop the dignity and autonomy necessary for voluntary societal engagement, the emphasis should currently be on young adults learning to integrate into social life. If young adults do not proactively develop their ability to navigate social systems, they risk exclusion. This study focused on youth with intellectual disabilities (YAwID) enrolled in special vocational schools who are preparing for social participation. Garden education functions as a social prescription that reveals individual potential and offers new chances [13]. Gardening activities can promote community participation and increase the vitality of YAwID. This can help bridge the gaps in social, mental, and physical health. Garden therapy is readily accessible to individuals with intellectual disabilities because it does not depend exclusively on verbal instructions in its pedagogical approach. The educational process entails engagement with instructors and cooperative endeavors with classmates, facilitating active involvement and autonomous tasks. Consequently, participants may experience a sense of familiarity rather than apprehension regarding the garden education assignments [14].

This research aims to investigate the consequences that arise when various individuals establish relationships and create and manage spaces utilizing gardens with varied characteristics. The traits of YAwID can differ significantly, ranging from minor to severe impairments. Despite extensive documentation of the traits of individuals with intellectual disabilities, it is crucial to recognize the uniqueness of individuals. The diversity in traits requires tailored strategies in education and assistance to enhance integration and quality of life for individuals with intellectual disabilities. We employed participant observation methodology to examine the cognitive, behavioral, and social dimensions of the individuals while assessing the treatment outcomes. Participant observation is a qualitative research method wherein the researcher immerses oneself in a community or social setting to observe and interact with participants. This approach is especially beneficial for comprehending social functions, cultural practices, and community dynamics. We employ qualitative approaches, including interviews, field diaries, and focus groups, to gather comprehensive qualitative data. [15]. This methodology prioritizes the involvement of participants and stakeholders throughout the study process, from data collection to dissemination. [16]. These methodologies can validate the direct efficacy of garden education and play a crucial role in substantiating the possibility of sustainable expansion. The findings provide important implications for identifying the efficacy and potential for sustainable growth through garden-based learning for YAwID and validating the impact of gardens as a therapeutic landscape.

### 1.2. Current Research

When people have a close relationship with nature, they feel emotional stability and pleasure, and their concentration improves [17]. Studies have confirmed that, in a therapeutic sense, natural spaces have different values and meanings than hospitals and homes. Stress decreases and positive emotions develop when people interact with nature and view plants, water, or gardens [18]. Natural spaces serve a special therapeutic role in facilitating a healthy life and forming relationships [19,20]. Thus, natural environments are prime examples of therapeutic spaces that help the treatment, recovery, and rehabilitation of patients in poor health [21]. Gardens serve as the most effective therapeutic element among various natural elements, significantly impacting our daily life. Gardens have a unique function as healing environments among the various components of nature. A garden is both a static pursuit, characterized by the observation of nature and the enjoyment of relaxation and leisure, and a dynamic endeavor, involving the selection of soil, sowing of seeds, and the actual planting and maintenance of flowers and plants [15]. The garden’s public accessibility enhances the proliferation of green welfare values among numerous residents [22]. Furthermore, the garden location can help integrate social, emotional, and symbolic elements that can deliver a lasting and vibrant experiential environment [23]. Private gardens, as ordinary places, become happy gathering places, redolent of friendships and support [24]. Gardens are places of comfort wherein diverse activities are performed and mental, physical, and spiritual healing is supported [25]. The therapeutic garden has helped individuals feel a decreased sense of loss, greater creativity, improved self-expression, and greater engagement in social interactions, and opportunities for sensory stimulation, improved self-esteem, and space for physical exercise [26]. They are also an effective way of improving the health and wellbeing of people of all ages [27].

Various studies on the therapeutic role of gardens for people with mental health issues have included older adults, youth, college students, office workers, and veterans [28,29]. However, this study focuses more on investigating the implications of gardening among young people for future education and the potential of gardening to enhance the functions of YAwID, aiming to help them adapt to the social environment and integrate into society.

Accordingly, it was necessary to understand the influences of gardens on young people. Green spaces enhance concentration and reduce stress in youth [30]. Moreover, they are reported to help reduce attention deficit, hyperactivity, and depression [31]. These findings indicate that gardens, through the familiar mediators of flowers and plants, can contribute substantially to improving mental stability and physical health in youth. This study’s participants possessed unique characteristics, which meant that we could not analyze the effects of gardens using common assessment tools. We employed the garden-based learning method—a practical educational approach—to examine the influences of gardens on YAwID. Based on previous findings that garden-based learning positively affected the academic performance and personal behaviors of students who had lost interest in learning [32,33], the present study deemed this approach appropriate for its goals. Yang et al. [34] conducted policy research of a garden-based therapeutic program implemented in collaboration with the Forest Service. The goal of this study was to investigate the viability and initial outcomes of a therapeutic gardening program during the COVID-19 pandemic. Despite the diversity of participants, they could not identify the impact of gardening on individual traits. Participants who had not been included in earlier research had to be chosen and given a particular focus. We addressed the limitations of previous studies by analyzing the phenomenon and therapeutic effects of garden-based healing and learning in more depth.

## 2. Materials and Methods

### 2.1. Implementing a Garden-Based Learning Program

The study recruited participants who did not face difficulties in basic communication and desired to lead a self-directed life by utilizing the knowledge acquired in the learning program. Because the program was long-term and focused on on-site training, participants needed to be able to work independently and effectively communicate with others. The students of Holt Special School, located in Goyang City, Gyeonggi-do, met these requirements. Founded in 1962, the school has operated various vocational programs for developing the potential of students to encourage independent living and enhance social adaptability. The school is mainly attended by young adults with mild intellectual disabilities, aged 20–21 years. They are suitable candidates for this study because of their high willingness to participate in social activities and gain basic communication skills, making it possible to assess the program’s effects.

A total of 26 students, including 13 men and 13 women, participated in this study. All these students were young adults, with an average age of 20.5 years. Due to their intellectual disability, they are unable to immediately engage in social activities and are therefore continuing their education in school. The study was conducted from July 13 to October 26, 2022. As the participants needed to express their thoughts and feelings, the recommendation of the teachers who were teaching students with severe disabilities was sought, and consent of the students’ guardians was obtained for their participation. The study was conducted by two gardening instructors, two garden designers, and a psychologist; the experts participated in every session of the educational program for efficient research. Prior to implementing the garden-based learning program and the validation and assessment of the research, the objectives and design of the study were explained to the participants, and data were collected from those who consented to participate. This study was approved by the Institutional Review Board of Korea University (protocol no. KUIRB-2022-0218-03; 5 April 2022).

Since the study participants were YAwID who have poorer capacity for adaptive behaviors and shorter attention spans than those without intellectual disabilities, common educational methods have not been shown to be effective. This study adopted an approach that would attract the attention of the participants and help them develop a sense of positive self-efficacy after participation. According to Burt et al. [35], field training develops students’ ability to apply knowledge to real situations and piques their interest. In particular, a nature-based outdoor program is a special approach to support students’ social, emotional, and intellectual improvements, including academic performance [36]. Such an approach could enhance awareness of sustainable life and environmental value while facilitating the immersion of the participants [37]. This study calls for a program wherein participants would feel less burdened, and positive or negative assessments could be made without hesitation.

**Figure 1:**
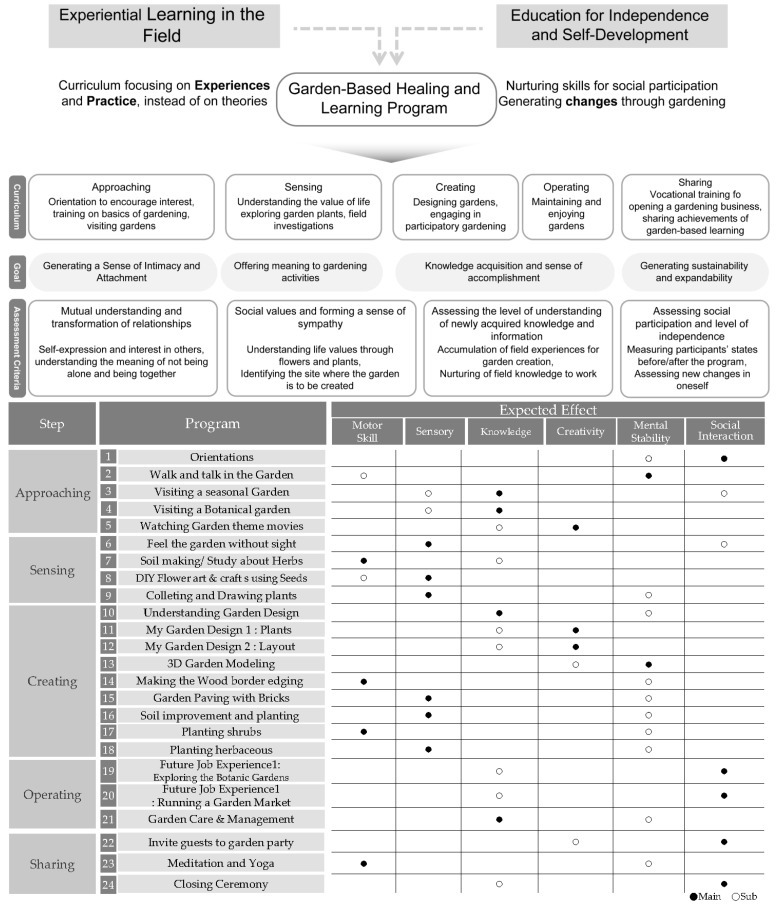
Garden-based healing and learning program.

This study developed a customized garden-based healing program wherein participants could develop diverse capabilities by acquiring self-help skills, enhancing self-determination, and improving social relationships. To participate, basic reading skills, arithmetic competence, and communication skills using limited language were required. Educational objectives were determined according to the characteristics of YAwID who possess insufficient adaptive abilities to integrate into society to help their social growth through garden-based learning. The program was designed with respective goals and expected effects (Figure 1). Over the course of three months, we planned and carried out a 24-session program that helped people heal by improving their motor skills, sensory abilities, knowledge, and memory, as well as their creativity, mental health, and social interactions. We observed every practice to see how well it reached the goals that were set. A total of 26 people attended each two-hour session.

The curriculum included understanding gardening, creating a garden, managing a garden, and gardening activities, each of which comprised approaching, sensing, creating, operating, and sharing. At each stage, participants were encouraged to achieve set goals, such as building intimacy and attachment, finding meaning, acquiring knowledge, enhancing feelings of accomplishment, and deriving sustainability and expandability.

The approaching step aimed to aid them in comprehending the garden and gaining fundamental knowledge. The participants strolled around the garden, talked, and visited it by season. The next step was to use your body and mind to experience the garden. A program was implemented that enabled participants to engage with nature through their five senses. Participants were able to make nutritious soil and embellish it with blossoms. The creating step involved designing the garden according to one’s desires. The preparation of the garden’s construction space and the planting of flowers created a unique experience. The operating step was a field training course in which students were responsible for the management and utilization of the gardens they had established. Participants were provided with vocational training to either operate a garden market or become a gardener. The final step is to practice yoga and have a party in the garden. The garden created by the participants was made to be a unique place that gives them a sense of accomplishment and attachment and a source of enjoyment.

As the participants found it difficult to sustain focus during theoretical lectures, the program focused on experiential activities that would arouse interest and encourage active participation. The program was operated flexibly, adjusting break time and class time according to the observed participant responses so that they could focus on the program. In addition, the educational content was designed to draw participants’ attention and enhance their concentration and engagement using elements of nature [35].

Each session was designed to allow the participants to explore and observe plants, express their feelings in the process, and handle and interact with the plants while learning gardening-related knowledge. The study intended to build a foundation by teaching YAwID to manage and maintain gardens, introducing them to diverse values and learning. Practical field training, rather than theories, was emphasized, and the program was largely aimed at cultivating social skills as a long-term therapeutic effect. Specifically, it aimed to impart knowledge on applying sustainable gardening in daily life by using the school infrastructure.

### 2.2. Data Collection and Analysis

This study collected systematic data through interview records and participant observation. By applying Spradley’s participant observation method [38], researchers can examine the social, psychological, and educational changes in people with disabilities, and analyze outcomes, such as social adaptability and mental stability, from diverse perspectives. The participant observation method can overcome the limitations of quantitative research based on statistical verification by helping interpret the changes evolving within the mind of the participants as the physical and psychological distance between the researcher and the participant decreases. However, it is not an objective research method that verifies changes using numerical values. Instead, it is a direct method of effect validation in which the researcher observes, communicates, and checks the growth of the participants and the changes they experience on-site.

The purpose of this study is to confirm the healing effects through personal changes before and after participation in garden education. First, the characteristics of the disabilities of each of the 26 participants were identified. This allowed us to ascertain their psychological state, attitude towards classes, social relationships, and future aspirations through pre-interviews with the teachers in charge. And during the three-month period of the training, three researchers were dedicated to observing the participants, and one psychologist conducted in-depth interviews to determine how the vulnerable areas had changed. The qualitative data generated through this process consisted of 624 participant observation records and 234 interview materials. Using a scientific approach to generate qualitative data is a key element of this study. The researcher who collected the data was an expert with extensive experience in analyzing research phenomena and a deep understanding of the data to be collected. The researchers tried to communicate comfortable and honest emotions, fully understanding the individual characteristics of the participants. Therefore, they were able to express their thoughts naturally.

**Figure 2:**
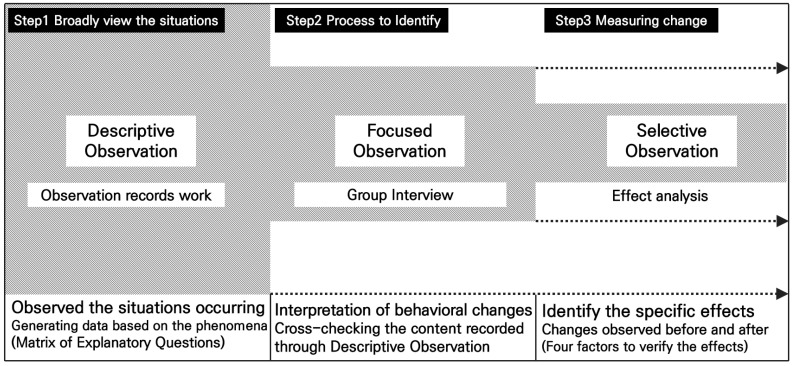
Participant observation method.

We employed this methodology by collecting and analyzing data using the three-stage process of descriptive observation, Focused Observation, and selective observation based on Spradley’s observation method (Figure 2). A researcher identified the situation and provided an objective evaluation to achieve the results. Therefore, we can assert that there is limited scientific trustworthiness. However, it can also be referred to as interpretive science because the results were reached after methodically analyzing observation and interview data collected by researchers stationed at the site for a long time.

First, it begins with the descriptive observation stage, which aims to enhance understanding of the participants and broadly view the situations arising through gardening. This process involves each researcher generating data based on the phenomena they observe, listen to, and experience. The researcher observed the situations occurring on-site and the changes in participants’ behavior, and recorded the participants’ actions, words, experiences, feelings, and spontaneous conversations. At this time, the Explanatory Question Matrix was used as a criterion for analyzing participants’ behaviors and reactions (Table 1). These questions were composed of seven aspects: space, elements, activities, time, participants, goals, and feelings. The observer closely observed and recorded the phenomenon using these questions. Seven factors—space, elements, activities, time, participants, goals, and feelings—were considered. The observer carefully observed and recorded the incident using these questions. This method is good for examining observation because it uses a long enough duration to avoid interpretive distortions. Summarizing with field notes during the program ensured speedy documentation, and four researchers analyzed the observation data thereafter. Participant observation helps explain each moment’s significance, direction, and relationships. The researcher deepened participant ties and fostered emotional bonds to elicit such outcomes.

Next was the Focused Observation stage, where in-depth group interviews with participants were conducted to verify and confirm the previously derived content. It was not merely a matter of recording and describing the situation that occurred on the site; rather, it was a process to identify what is inherent in it. The interviews took place every Tuesday from 10:30 to 11:30 a.m. during program operations for nine sessions. This course was administered by a team of psychologists and observation researchers, who meticulously documented their findings and divided the 26 participants into 2 distinct groups. The focus group interviews were executed using a method that involved cross-checking the content recorded through descriptive observation. Through interviews with 26 people, we were able to see what actions and changes had occurred in comparison to the participants’ existing characteristics (Table 2). The interview had two questions: the first was “please narrate the most significant experience in your gardening education”; second, “please explain the effect that garden education has had on you”. The purpose of this is to determine whether the participants had positive or negative responses and to identify the factors that influence them. The following phase was based on the findings of these interviews. The researchers instructed the participants to speak freely about the experiences and effects they had confirmed during the gardening, and they had to identify the aspects that matched what they had observed. The final descriptive result was considered trustworthy if the participant’s experience and the research team’s observations were consistent.

The final step was selective observation, which generated results by applying selective criteria to previously analyzed data. This procedure involves selectively checking significant meanings by thoroughly analyzing the extensively identified content from the prior stage. Initially, it is necessary to comprehend all of the situations that happened during the garden-based learning program. By identifying the meaning inherent in the situation, we analyzed the positive or negative changes that had occurred. Prior research has verified that garden therapy has personal, social, physical, and mental effects. Therefore, this investigation implemented it as a selective observation criterion. The primary objective of this study is the kind of healing effect that garden learning generated for the individuals who participated. In order to compare the changes before and after, we conducted a more thorough examination of the personal changes identified in Focused Observation. Accordingly, the garden’s therapeutic properties were classified into four categories, with specifics on each healing effect organized (Table 3). The conclusion provides an explanation of each aspect of the healing effect.

## 3. Results

This study investigated the relationship between therapeutic landscapes and learning. Specifically, among numerous types of therapeutic landscapes, this study examined gardens as a place for learning and its effects on YAwID’s self-growth and improved adaptability to environmental changes. The results showed that the garden-based learning program helped YAwID build social relationships, improve their physical and mental wellbeing, and nurture their social skills. Gardening activities motivated positive thinking, expanding the scope of interest and attention and reinforcing physical, mental, and social restorative capabilities. The characteristic of garden-based learning programs is that they induce emotions such as interest, joy, pleasure, and happiness while participants engage in productive activities handling plants and interacting with nature, thereby enabling emotional therapy. In the process of learning and growing accustomed to the repetitive task of growing and caring for plants, participants were able to enjoy the social therapeutic effects of gardening, such as acquiring professional knowledge, feeling a sense of professional accomplishment, and developing capabilities for vocational rehabilitation. Hence, the program helped in recovering psychological stability, building social relationships, and improving individual capabilities.

### 3.1. Emotional Healing for the Recovery of Psychological Stability

Being close to a garden provides an opportunity to share one’s feelings with plants as people find links between the lifecycle of plants and their own. Thus, caring for plants has a positive influence on those who cannot live a self-directed life due to their lack of self-determination and provides a sense of responsibility and accomplishment.

*Planting flowers and lugging soil around with my friends made me sweat a ton, but it felt really good. It’s funny how hard work can actually make you feel great. Watching the plants grow and thrive helped calm my anxiety. When I saw the garden changing, I could feel the mystery of life. I became confident and thought that I could also make it. I think the garden gave me happiness.*

*(Participant* *M)*

The garden-based learning program focused on providing participants with the opportunity to create a healthy and safe space by and for themselves. Participants could create a space wherein they could escape from a daunting society and feel comfortable and safe without help from others. In this space, they enjoyed private time or interacted with friends, unknowingly experiencing the positive influences of the outside environment.

*Throughout the program, the participants were so happy simply because they were in the garden; not in the class. They talked with friends and sometimes sang a song in the garden they made by themselves. We hold cultural events, such as aerobics and flea markets. It seems that now this place has become a playground and a shelter, especially for the youth. *

*(Instructor* *A)*

### 3.2. Social Healing Through Social Relationships

Garden-based learning can build social skills and self-esteem in people with interpersonal difficulties. Participants developed a sense of understanding and cooperation as they worked together to solve challenging tasks in the garden, which became a common interest. It had a positive effect on connecting “people to nature” and “people to people.” The garden sensitivity training course helped participants develop an appreciation for nature by engaging all five senses with garden materials. The garden trip allowed them to explore plants, learn from them, and create precious memories together.

*I still chat with my friend who held my hand when we were learning to close our eyes and feel the texture of the plants. Planting flowers and pulling weeds is tough but I have great memories of doing it with my friends. We bonded through all the hard work and got close enough to share our problems.*

*(Participant* *K)*

Through gardening, patience and concentration can be developed. In particular, people develop the ability to accept the emotions of others to maintain a collaborative relationship. Gardening encourages a positive way of thinking, be it a sense of stability, patience, or respect.

*A participant did not take part in transporting seedlings so others yelled at them until they cried. However, after realizing that the participant was allergic to pollen, the others showed an act of caring by allowing a short break time. They also understood the situation of a participant with an injured arm and told them to rest and not feel sorry about it.*

*(Instructor* *B)*

What initially felt awkward and uncomfortable turned into something positive. Fond memories remain of the people they met during the garden training, the friends made, and the guests invited to the finished garden. The positive effects of incorporating the garden into the program have grown. Participants who relied on assistance took ownership of the garden and created special memories. The garden became a medium for social interaction.

*Towards the end, I started feeling sad. I hated saying goodbye to the teachers I met through the class. I remember all the fun times with my friends, creating the garden, having garden parties, and inviting guests. The memories we made during the garden class are really precious to me.*

*(Participant* *U)*

### 3.3. Physical Healing Through New Activities

As gardens were used as a place of learning, students who usually did not participate in physical activities were encouraged to come outdoors. Touching soil and planting flowers made it possible for students to be with nature, develop a sense of space, and contribute to joint movement and activation of muscles. Acts of digging and laying soil were helpful for gross motor skills, whereas planting flowers and seedlings and trimming roots, stalks, and leaves improved fine motor skills.

*Compared to the theory lectures in class, it was a special experience to come out and dig soil, plant seeds, and sweat with friends. For the first time in my life, I used a garden trowel, planted flowers, and pulled out weeds, and I moved heavy packages, cut timber, and made wood fences. At that time, it was hard for me to do such things repetitively but I felt comfortable and rewarded when I saw flowers and plants growing.*

*(Participant* *D)*

With flowers and plants functioning as mediators, gardening arouses people’s five senses while improving cognition. Furthermore, various cognitive activities performed in gardening stimulate the senses, improve immunity, and activate the circulation of blood and hormones, improving overall health. The results of this program show that gardening actively changes participants.

*Sometimes I walk around the garden. I smell the plants and see how tall each plant has grown, calling their names. In particular, I wonder whether the flower I planted is growing well and it is marvelous to see them change as time goes by. I can occasionally see small bugs that I only see in books. When I’m out in the garden with my friends, hearing the birds chirping around me, it helps me clear my head more than listening to music.*

*(Participant* *E)*

### 3.4. Independent Healing Based on the Ability to Be Different

Typically, YAwID have difficulties maintaining interpersonal relationships. However, through gardening, they naturally learn and acquire sociability by sharing outputs with others, recognizing the value of their existence. This indicates that gardening is an effective tool to encourage the social participation of people with disabilities and improve their quality of life.

*It was a marvelous experience, seeing the garden exactly as we had imagined in front of our eyes. Referring to the picture that we first drew, we planted flowers one by one and spread soil, and a beautiful garden was finally created. I feel so proud of the garden because we created it with friends. Specifically, I really want to show the flower that I planted by myself to my parents.*

*(Participant* *Z)*

Through the garden-based learning program, some participants identified their areas of interest and attention and expressed delight after completing the garden. When people design a garden according to their interests, they feel emotionally stable and achieve a sense of accomplishment.

*The participants found their work rewarding as they watched the garden come together. They enjoyed activities, such as loosening the hard soil, cutting wood, moving supplies, digging, and planting flowers. Their growing interest in gardening made them aspire to become gardeners.*

*(Instructor* *C)*

Garden-based education shows the potential to evolve into a new vocational rehabilitation. YAwID who are interested in flowers and plants can explore new career paths as they discover various interests and capabilities. They desire to overcome their limitations by learning skills through repetitive training and accumulating experiences of success. This means that garden-based learning refines emotions in the process of caring for plants and promotes occupational self-achievement.

*One-time programs give pleasure and joy for a short time but soon fade from memory. Consistent healing for youth with intellectual disabilities means helping them make a wise and appropriate decision based on sharp observation of the problems in the field and having pride in themselves while working in society. I would like to lead the students with an interest in gardening into becoming social gardeners.*

*(Instructor* *D)*

## 4. Discussion

This study explored the effects of garden-based learning programs on the knowledge, skills, and attitudes of YAwID. Participants directly observed plants growing, felt emotionally supported, and built positive relationships through collaborative activities. Moreover, while gardening, they acquired the knowledge, skills, and attitudes required to make responsible decisions and the ability to effectively apply them. The program confirmed the effects of gardening that were observed in previous studies, including stress reduction and blood pressure improvement through contact with the natural environment [39], improvement in everyday mindset [40], relief from social isolation [13], lowered anxiety [41], and immunologic effects against stress [30]. The reasons for these effects were that the participants became aware of the importance of life while sowing seeds and growing plants and found value in their existence. In the garden, they communed with living things, such as flowers, plants, and insects, and, in the process, felt happy. Furthermore, as the garden acted as a living lab, participants learned and accumulated hands-on knowledge. In other words, gardening provided new knowledge to those with difficulties living in society due to low intelligence and lack of comprehension. Participants engaged in self-directed activities and felt a sense of accomplishment. They learned and developed an altruistic attitude through the program. YAwID usually have very low self-esteem and are helped and cared for by others. However, in the process of growing plants, they developed the confidence that they could care for others. This change in the mindset improved social, emotional, and cognitive effects. The garden-based learning program improved the psychological state of YAwID and offered them the opportunity for vocational rehabilitation. Repetitive cultivating activities involved in growing and caring for plants enhanced specialized skills. The process of regulating emotions through gardening extended their self-efficacy. Thus, gardening had a direct impact on the promotion of health and social independence.

Garden-based participatory learning induces direct and active experiences. While in contact with nature, people have positive emotional experiences, and activities focused on participants’ interests heighten their concentration to allow for the accumulation of new knowledge. Horticultural therapy is appropriate for YAwID because, rather than being dependent on verbal instructions, it provides alternatives to interact with instructors and participate in collaborative, active, and self-directed activities with peers. This is enhanced when participants voluntarily join gardening activities and enjoy the tasks without anxiety or fear. In addition, considering the characteristic of YAwID of being dependent on others’ thoughts and opinions, the experience of touching and handling real objects can lead to emotional stability and improved social adaptation and physical motor skills. Gardening facilitates the promotion of physical and mental health and becomes an instrument for the rehabilitation of participants’ disability. In other words, gardens are a space for facilitating social relationships, promoting healthy growth and development, contributing to physical activities, achieving a positive mindset and mental happiness, raising cohesion in communities, and nurturing solidarity in neighborhoods.

Based on the findings, garden-based healing programs can serve as an alternative public health service for YAwID. Traditionally, medical interventions, such as psychotropic drugs, have been used to supplement the capabilities people were deemed deficient in. However, more recently, the social model that posits that people with disabilities can live as members of a community is gaining attention and importance [42]. The social model is closely related to living in a family and with others in the local community, which is emphasized in disability welfare. Thus, sustainable care for the disabled includes them living as members of the local community and society by their abilities. Particularly, employment is an important process for earning income and for an individual’s existence to be recognized and valued in society. In this context, garden-based learning programs can work as a social model for healing for YAwID.

The findings showed that the health-promoting effect of therapeutic landscapes is enhanced and reinforced when people directly participate in the activities. The wellbeing effects of green spaces, fields, and parks [43] are maximized when people’s experiential activities are added. This can be explained by the attention restoration theory, which states that the surrounding environment affects a person’s life [44]. Nevertheless, the natural environment has a positive restorative effect [39]. Depending on environmental characteristics and human behavior, the positive and negative effects can vary [45]. Differences in perception arise with variations in conditions, such as accessibility of the topography, climate conducive to long-term stay, relationship with others, and the amount of knowledge and information obtained from the place [20]. A characteristic of gardening confirmed in this study is that of adding experiential activities to the natural environment. In other words, when active participation through education is achieved in a pre-existing natural environment, the space gains greater value. Such phenomena arise when therapeutic landscapes and humans interact vibrantly to create a therapeutic experiential landscape. Therapeutic experiential landscapes can be seen as an extension of therapeutic landscapes and are characterized by maximizing therapeutic restorative potential when mediating activities (e.g., learning, enjoying, and playing) are performed in a pre-existing natural landscape. This study was conducted in the specific space of gardens; however, there is a need for further research on the effects of experiential interventions on different types of therapeutic landscapes.

## 5. Conclusions

This study investigated the healing effect of gardening on YAwID. As it is not easy for YAwID to collect information, experience situations, and make decisions independently, we had to devise an approach that could help the participants utilize the garden’s resources properly. Consequently, we developed and implemented a customized learning program for YAwID and analyzed its impact. The findings showed that the garden-based learning program transformed participants’ perceptions of disability and cultivated their ability to respond to challenges. It was found that those who initially had negative emotions such as passivity, negativity, fear, and discomfort before participation showed greater positive effects after participation such as a sense of stability, respect, mutual interaction, skill improvement, and increased expressiveness. This ultimately facilitated the reinterpretation of the value and significance of garden-based healing and the derivation of the central effects of this approach.

Participants experienced sympathizing with others; maintaining positive relationships; and obtaining the knowledge, skills, and attitudes required for behaving responsibly through gardening. Garden-based learning, utilizing flowers and plants, enhanced the physical and mental health of YAwID, improved their functioning, and helped them adapt to the environment and integrate into society. As a form of vocational training by which people with disabilities could become employable by learning gardening skills, the program helped promote their participation in society and upgrade their quality of life.

This study used a qualitative research method as a method of verifying the effects of garden therapy. This approach was implemented to capture the complexity of human experience, which is difficult to verify through quantitative research. These methodologies are particularly effective in exploring culturally sensitive topics, understanding individual experiences, and examining process-based research questions. This study sought to identify the nuanced emotional shifts in participants with intellectual disabilities through the complex phenomenon of garden therapy. Participants shared their experiences through post-program interviews, which also revealed the healing effects. They realized they experienced various changes during the program, such as unusual inspiration, special contributions, and a sense of achievement and satisfaction. Through this observation and analysis, the specific determinants of change as well as their meanings and perspectives can be identified. However, the research raised issues such as data reliability, subjective interpretation, and limited generalizability compared to quantitative methodologies. We fully utilized the strengths of qualitative research methods to analyze the effects in detail. This study confirmed that qualitative research methods are a meaningful attempt. The preliminary and exploratory analysis of the garden’s therapeutic effects on young adults produced significant findings. Nevertheless, it is important to recognize the study’s limitations and incorporate additional measures to guarantee dependability and accuracy in subsequent studies.

## Figures and Tables

**Table 1:** Matrix of descriptive questions.

	Space	Element	Activity	Time	Participants	Goal	Feeling
**Space**	Could you provide any information about the location of the garden?	How are the garden elements organizing the space?	How can I arrange my garden space to promote activity?	What is the garden’s evolution over time?	Which space of the garden do participants prefer?	What do you want to achieve with your garden?	Which garden spaces have influenced your feeling?
**Element**	Where are the garden elements situated?	Can you describe in detail all the garden elements?	Which garden elements are used in activities?	How do garden elements change over time?	How do participants use garden elements?	What garden elements contribute to achieving your goals?	Which garden elements have influenced your feeling?
**Activity**	Where are the garden education activities occurring?	What garden elements affect activity?	Can you explain the activities that occur in the garden?	How does the garden’s activity evolve over time?	What is the process for participants to engage in garden activities?	What activities were undertaken to achieve the goal?	Which garden activity has influenced your feeling?
**Time**	Where may one observe the alteration in time?	What elements of the garden make you sense the passing of time?	What are the hours for gardening activities?	When can I experience the passage of time in garden education?	How much time do I need to participate in the garden training?	What is the relationship between objectives and time?	When did your emotional transition take place?
**Participants**	Where do participants receive garden training?	Which garden elements are of interest to participants?	How do participants engage in garden activities?	How do the participants perceive the passage of time?	Describe your participants’ characteristics?	What do participants do to achieve their goals?	What causes your feelings?
**Goal**	What kind of garden do you need to create to achieve your goals?	What garden elements influence your healing objectives?	What activities did you undertake to achieve your goals?	What is the relationship between goals and time?	What goals do participants want to achieve?	What are your objectives for this learning?	Have your emotions evolved to align with your therapeutic goal?
**Feeling**	Where do the various feeling states occur?	What feelings lead to the use of what objects?	What are all the ways feelings affect activities?	How do changes in emotion relate to changes in time?	Can I really comprehend the emotional shifts of the participants?	How did the change in emotion affect your goal?	What emotional shifts have occurred?

**Table 2:** Focused Observation findings.

No.	Name	Characteristics	Observation
1	A	Emotionally sensitive, and unable to interact with friends	A seems to have made new friends with flowers, bees and butterflies while participating in gardening.
2	B	Difficulty presenting because they are not adept at communicating their emotions to others	When B was being educated indoors, she did not show any smile, but I saw B chatting with friends while planting flowers outdoors.
3	C	Inadequate interpersonal relationships because of passive-aggressive behavior	C had a hard time keeping close to his friends, but C actively helped with the heavy work of carrying loads and digging up soil.
4	F	Actively involved in training but quite individualistic	F used to focus on his work alone when he was receiving indoor training, but when F was creating a garden outdoors, F would ask his friends for help.
5	L	Lack of empathy and an inability to articulate one’s own experiences	Every time the class ended, we would talk about how he felt about the day’s lesson and L would share feelings like “Teacher, I felt good today” or “Teacher, I had a hard time today” without me even having to ask.
6	O	Limited cognitive capacity and passivity in social interactions	O passively participated in the program without any significant changes, but O was a student who attended without any absences until the end.
7	S	Have normal intelligence yet lack empathy for others.	S preferred to enjoy the program alone rather than socialize. When teachers intentionally gave S heavy tasks to do together or missions S could do with friends, he acted very awkwardly but did not refuse.
8	D	Negatively reacting to circumstances that do not align with one’s expectations	D couldn’t concentrate when he attended lectures indoors, But the amazing thing is that D never expressed any complaints during the garden program. D was a participant who was very curious about garden plants, how to make them, and so on.
9	I	Likes doing things outside with their hands and has trouble managing their anger	I expressed dissatisfaction when something didn’t go as planned in the gardening activities. However, when I was shoveling and turning the soil repeatedly while sweating, I silently focused on the task at hand without saying a word.
10	Q	Likes to be with people and actively participates in everything	Q became very interested in creating gardens and wanted to invite her friends and parents to see them. Q also expressed her desire to become a gardener if he had the opportunity.
11	R	Positive and highly engaged, but struggles with emotional regulation	R was in charge of the most difficult task in the process of creating the garden. R seemed to be about to get angry, but his mood seemed to improve quickly with the compliments of friends.
12	V	Cognitive impairments, leading to inadequate learning and emotional fluctuations	V had a low understanding of individual lessons, but his concentration in team-based lessons was high.
13	Y	Trouble getting along with friends and not being able to control yourself	Y still struggled with interpersonal skills, but he enjoyed the gardening program.
14	E	Proficient verbal abilities and knowledge, yet inadequate expression stemming from nervousness	E is shy, but E knows how to express E’s feelings to those E has become close to, and sometimes E is often seen enjoying the garden alone.
15	K	Desires acknowledgment from peers yet is apprehensive about little errors.	K showed enthusiasm during class time by actively participating in completing the project through collaboration, and K also expressed interest in helping with the work of other teams K wasn’t a part of.
16	P	Not being able to communicate effectively and not being heard	P still struggled with interpersonal skills, not interested in gardening
17	U	Possesses musical aptitude, however, finds it challenging to communicate it to others	On the day of the event program, I asked U to sing the song U performed in the garden, and U sang it confidently, receiving applause from all the kids in the class. That day, U looked very proud.
18	G	A high level of cognitive ability, but no class feedback is provided due to a lack of learning ability	G was more active in extracurricular classes than in regular classes and G found studying gardening enjoyable.
19	H	Despite having a cheerful outlook on life, although struggles with resolving intricate issues	H still found it difficult to start solving problems, but during the program, H made a lot of effort to try with friends or teachers.
20	J	Having trouble speaking because of delayed language development	Verbal communication was difficult, but the gardening practice have given J to confidence.
21	N	Lack of emotional expression because of aberrant language development	N is still clumsy at expressing his emotions, but in the latter part of the class, N started to talk with his friends.
22	Z	Lack of emotional control and abnormal language development, but eager to learn	Around the time the program was ending, Z wanted to become a gardener and earn money.
23	T	Possessing an average level of cognitive function, but lacking in empathy for others	T participated passively until the middle of the program, but in the latter part, T showed significant changes by helping friends and actively giving presentations. T also requested to be introduced to other good gardens, expressing a desire to visit them.
24	W	Inadequate communication resulting from insufficient linguistic proficiency	W still struggled with interpersonal skills.
25	M	Provides numerous viewpoints and demonstrates leadership among peers.	M found great joy in creating a garden with his friends. He was especially happy to watch the plants grow.
26	X	Possessing a positive disposition and fostering positive relationships with peers	X enjoyed gardening while helping the teacher and friends throughout the program.

**Table 3:** Selective observation findings.

Categories	Effects		
	Before		After
Emotional	Self-criticism and anxiety over failing Passive and negative behavior Dissatisfaction expressed on society	▶	Improved emotional stability and self-esteem Weakening of aggressive feelings toward others Enhance empathy and promote sensitivity
Social	Individualistic thought and selfish behavior Having trouble collaborating with others Absence of motivation to engage in learning	▶	Strengthen capacity for teamwork Enhance interpersonal skills with colleagues Enhanced social interaction in sharing and caring
Physical	Participating in indoor learning activities Lack of physical health promotion activities Deficiency in cognitive capacity	▶	Increase physical activity by spending time outdoors helps to activate muscles and joint movement Improving cognition through sensory stimulation
Individual capability	Not knowing what the future holds Concerns with social life Insufficient self-expression	▶	Occupational rehabilitation to accomplishment Improve judgment and decision-making Developing creativity and self-expression

## Data Availability

The data used in this study are not publicly available owing to the ethical restrictions imposed by the Institutional Review Board; however, they are available with the consent of the participants on reasonable request.
